# Factors Influencing Visits to the Pediatric Emergency Department

**DOI:** 10.7759/cureus.51995

**Published:** 2024-01-10

**Authors:** Hussain A Al Ghadeer, Jalal K Aldandan, Jawad S Alnajjar, Mohammed H Alamer, Saja A Almusallam, Abdulelah A Alneamah, Saba’a A Alnasser, Juwdaa S Al Najjar, Rawan M Aldihnayn, Najla R AlRashed

**Affiliations:** 1 Pediatrics, Maternity and Children Hospital, Al-Mubarraz, SAU; 2 Pediatrics, King Faisal University, Al-Hofuf, SAU; 3 Pediatrics, Al-Jafar General Hospital, Al-Jafar, SAU; 4 General Medicine, Al-Ahsa Health Cluster, Al-Hofuf, SAU

**Keywords:** pediatric emergency department, saudi arabia, urgency of illness, nonurgent visits, ambulatory care, emergency department

## Abstract

Introduction

Overcrowding in emergency departments (EDs) is still a national and international issue. Among the variables contributing to this crisis are an increase in patient numbers and the severity of sickness. One significant issue that has not yet been addressed and is burdening healthcare facilities is the use of EDs by parents of children who have mild illnesses. Developing successful interventions requires an understanding of the factors that lead to nonurgent visits to pediatric EDs (PEDs). Our objective was to assess the variables that could influence parental visits to PEDs.

Methodology

In the Eastern Region of Saudi Arabia, between September and November 2023, a descriptive cross-sectional survey was conducted among parents who had previously visited a PED. The survey had 21 questions. Along with parental viewpoints and healthcare utilization, parents’ evaluations of their child’s emergency state were investigated. In addition to gathering and evaluating demographic data, the survey evaluated respondents’ impressions of the severity of a disease or injury.

Results

A total of 776 participants were included in the study. The mean parental age was 32.1 ± 12.7 years, and approximately 32.1% of the participants’ children were between the ages of 1 and 5. Nearly half of the children, 44.7%, visited the ED during the evening shift. The most common reasons for presenting were fever (50.5%) and upper respiratory tract symptoms (37.1%). Among these visits, parents perceived 48.5% as nonurgent and 30.2% as urgent. The majority of respondents (54.9%) had received advice before going to the ED. In most cases (47.9%), this was from a relative or a healthcare provider (16.7%).

Conclusion

This analysis identified some of the reasons parents bring their children to the ED for mild illnesses. The results emphasized the varied nature of the problem. Understanding the reasons for parental ED visits may help us better design targeted interventions to decrease unnecessary visits and lessen the burden on healthcare systems.

## Introduction

The emergency department (ED) is one of the most important parts of the healthcare system [1]. Patients of all ages can receive urgent medical care in the ED on their own initiative. However, for newborns and small children, it is the parents who decide if they need urgent medical assistance [2,3]. Pediatric patients are still routinely treated in the ED, regardless of their primary medical complaint’s seriousness or acuity [4]. Earlier research found that nonurgent cases comprise 58% and 82% of pediatric ED (PED) visits, leading to inefficiencies, overcrowding, and negative impacts on the healthcare system [5-7]. Furthermore, 80% of babies are either discharged from PED or from the hospital within 24 hours, raising the question of the current service provision’s appropriateness [8], despite the fact that a primary care physician (PCP) is evaluating the majority of nonurgent medical issues [1]. A prior systematic review found that factors contributing to nonurgent ED use include younger age, convenience in comparison to alternatives, a physician’s referral, and negative perceptions about alternatives like PCPs [9]. In a previous study, the probability of using either a general practitioner or an ED was found to increase as the distance between the child’s home and that location [10]. Additionally, children from families with a lower socioeconomic status exhibited a higher tendency compared to other children to initially seek care from primary healthcare physicians [11]. In PEDs, unscheduled return visits and overcrowding reflect the inappropriate use of EDs. This inappropriate use has several negative effects, including longer wait times, higher healthcare system costs, limited time slots for individual patients, the need for multitasking, and frequent interruptions [12].

Saudi Vision 2030 identified the burden EDs experience. A 2019 study found that the PED is the main point of entry for medical care in Saudi Arabia, partially because all Saudi Arabian citizens have the right to receive comprehensive medical care without incurring any expenses [13,14]. Therefore, our aim is to determine factors influencing visits to the PED in Saudi Arabia.

## Materials and methods

Study design and selection criteria

This was an observational cross-sectional study conducted from September to November 2023 using an online questionnaire distributed among parents of children who were younger than 14 years, who were living in the Eastern Province, and who had a history of visiting the PED. However, parents of children who were older than 14 years, who had not visited the PED, and who did not agree to participate in the study were excluded. The approximate sample size was 385 based on the formula n = Z2 pq/E. The margin of error (E) equaled 0.05, and the confidence level (Za/2) was 95%. The population of the Eastern Region is 5.14 million, based on the general authority of statistics [15]. A randomized sampling technique was used to collect the data.

Questionnaire design and validity

We developed the questionnaire based on previous studies [16]. Then, we examined its validity by conducting a pilot study of 30 participants. The questionnaire was administered from September 2023 to November 2023. It was written in Arabic and consisted of three parts. The first part obtained participants’ informed consent. The second part contained different questions about participants’ ages, their children’s demographic data and their children’s condition, the time at which participants visited the ED, and the person who accompanied their children to the ED. The last part contained questions about various factors that influenced participants’ visits to the PED.

Ethical consideration and statistical analysis

An informed consent form was provided to participants before conducting the study. The data collected were kept confidential and used solely for research purposes, adhering to ethical guidelines for research involving human subjects. The Ethics Committee of King Faisal University approved this study (ethical approval code: KFU-REC-2023-OCT-ETHICS1249).

Data analysis

The data were collected, reviewed, and then fed into SPSS Statistics version 21 (IBM Corp. Released 2012. IBM SPSS Statistics for Windows, Version 21.0. Armonk, NY: IBM Corp.). All the statistical methods used were two-tailed, with an alpha level of 0.05. A p-value less than or equal to 0.05 was considered significant. Descriptive analysis was conducted by prescribing frequency distributions and percentages for study variables, including participants’ data, child data, ED visits, and factors influencing PED visits. __ reported baby problems that caused parents to visit the ED. Reasons that parents went to the ED and the degree of parents’ confidence that they could look after their child when the child was sick/injured were graphed. Cross-tabulation to determine the relation between a baby’s condition and factors of ED visits was conducted using Pearson’s Chi-square test. A probability test was conducted for small frequency distributions.

## Results

As shown in Table 1, a total of 776 eligible parents completed the study questionnaire. Parents’ ages ranged from 18 to 60 years with a mean age of 32.1 ± 12.7 years. A total of 249 (32.1%) participants reported that they took their children aged one to five years to the ED, 192 (24.7%) reported that they took their children aged six to 10 years to the ED, and 162 (20.9%) reported that they took their children aged one to 12 months to the ED. A total of 430 (55.4%) children visiting the ED were female. A total of 347 (44.7%) children visited the ED from 3 pm to 7 pm, 233 (30%) children visited the ED from 7 am to 3 pm, and 196 (25.3%) visited the ED from 11 pm to 7 am. Regarding who accompanied the child to the ED, most parents accompanied the child together (38.7%), followed by only the mother (27.6%) and only the father (23.5%). A total of 476 (61.3%) participants mentioned that they had taken their babies to the ED before, and 474 (61.1%) mentioned that they had brought any other child to the ED before.

**Table 1:** Bio-demographic data and ED attendance with the baby among study parents

Bio-demographic data	No	%
Age in years		
18-24	95	12.2%
25-30	185	23.8%
31-45	324	41.8%
>45	172	22.2%
At the last visit to the ED, how old was your child?		
<1 month	94	12.1%
1-12 months	162	20.9%
1-5 years	249	32.1%
6-10 years	192	24.7%
>10 years	79	10.2%
Child gender		
Male	346	44.6%
Female	430	55.4%
What time was it when you visited the ED?		
7 am - 3 pm	233	30.0%
3 pm - 11 pm	347	44.7%
11 pm - 7 am	196	25.3%
Who was with the baby in the ED?		
Parents together	300	38.7%
Mother only	214	27.6%
Father only	182	23.5%
Grandfather or grandmother	70	9.0%
Brother	4	.5%
Mother and brother	3	.4%
Sister	2	.3%
Mother and uncle	1	.1%
Have you brought this baby to the ED before?		
Yes, came before	476	61.3%
No, first time	300	38.7%
Have you brought any other child to the ED before?		
Yes	474	61.1%
I have only 1 child	114	14.7%

Table 2 shows parents' attitudes and perceptions regarding their children’s health status in the Eastern Region of Saudi Arabia. A total of 44.8% of participants agreed that their baby fell sick more easily than other babies, and 39.4% agreed that their baby was more fragile than other babies. A total of 94.1% of participants agreed that their baby was as healthy as other babies, and 31.2% reported that their baby had a long-term health condition.

**Table 2:** Parents' attitudes and perceptions of their children's health status, Eastern Region, Saudi Arabia

Attitude	Strongly disagree		Disagree		Agree		Strongly agree	
	No	%	No	%	No	%	No	%
My baby is as healthy as other babies	15	1.9%	31	4.0%	179	23.1%	551	71.0%
My baby is more fragile than other babies	265	34.1%	205	26.4%	185	23.8%	121	15.6%
My baby gets sick more easily than other babies	186	24.0%	242	31.2%	226	29.1%	122	15.7%
My baby has a long‐term health condition	345	44.5%	189	24.4%	128	16.5%	114	14.7%

**Figure 1:**
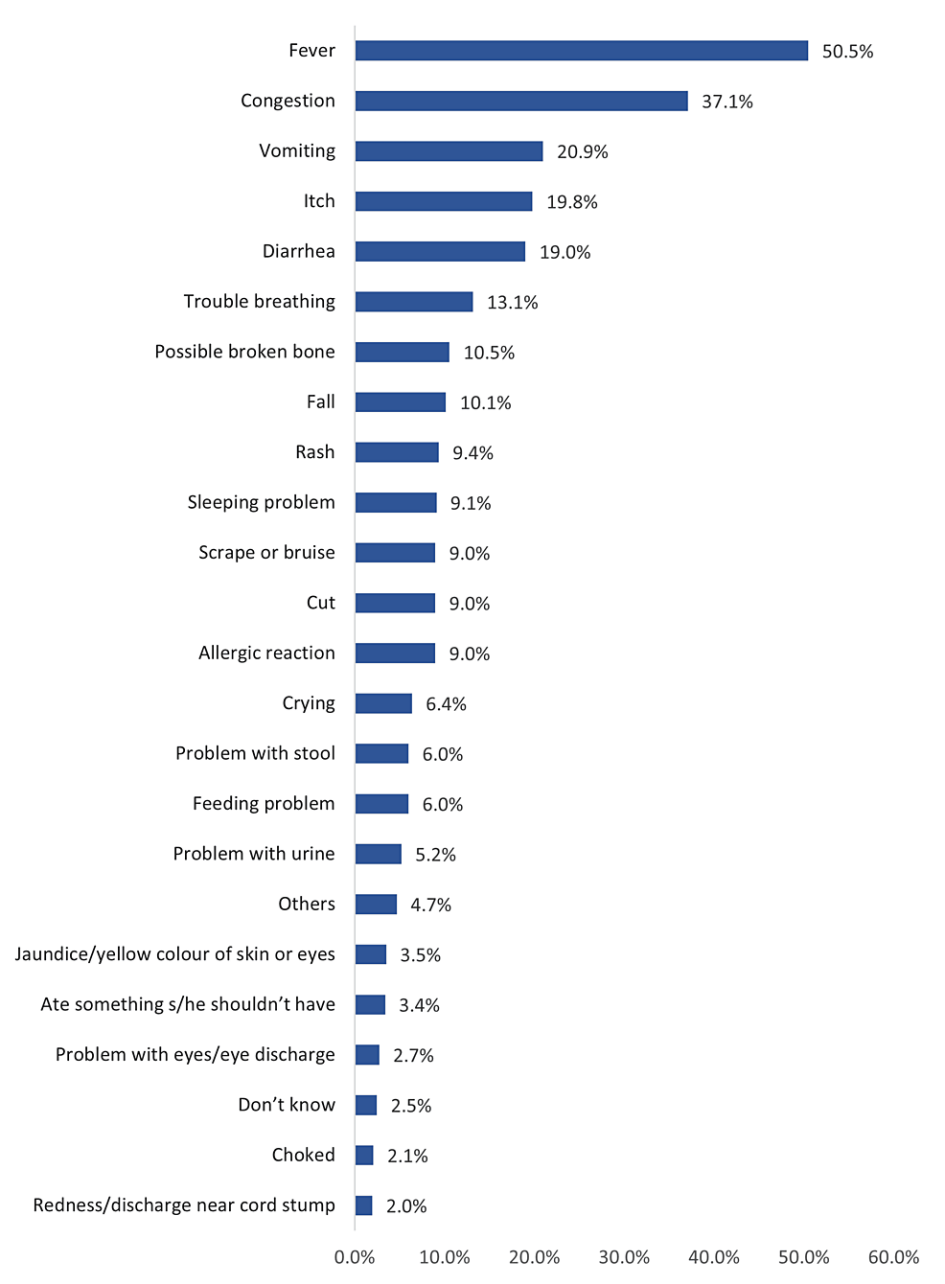
Reported baby problems that caused parents to visit the ED, Eastern Region, Saudi Arabia

Figure 1 lists baby problems that caused parents to visit the ED in the Eastern Region of Saudi Arabia. The most reported reasons were fever (50.5%), congestion (37.1%), vomiting (20.9%), itching (19.8%), diarrhea (19%), breathing problems (13.1%), possible broken bones (10.5%), and falls (10.1%).

Table 3 shows the factors that influenced visits to the PED in the Eastern Region of Saudi Arabia. A total of 376 (48.5%) of parents reported that their child’s problem was not very serious, but 30.2% reported it was serious. Additionally, 21.9% felt that something bad would happen to their baby in the next 24 hours if a doctor did not see the baby, and 24% said this was probable. A total of 500 (54.4%) parents tried medication and treatment at home for their baby’s illness or injury. Regarding the expected waiting time, 21% believed they would receive care immediately, and 43.4% believed they would receive care within an hour. A total of 426 (54.9%) parents got advice from someone before coming to the ED. This person was mainly a relative, friend, or neighbor (47.9%); the baby’s usual doctor (16.7%); office staff or a nurse at the doctor’s clinic (12%); or a pharmacist (11.7%). The most common bit of advice was to wait and watch the baby to see if the baby’s condition grew worse (28.6%), to bring the baby to the ED (25.4%), to treat the baby at home (17.4%), and to call the baby’s usual doctor (16.7%).

**Table 3:** Factors that influence visits to the PED as reported by parents, Eastern Region, Saudi Arabia

Factors		Count	Column N %
To what extent was your baby’s problem serious?	Not very serious	376	48.5%
	Serious	169	21.8%
	Very serious	73	9.4%
	Not sure	158	20.4%
In your opinion, would something bad have happened to your baby in the next 24 hours if a doctor did not see him/her?	Yes, definitely	170	21.9%
	Yes, probably	186	24.0%
	No, probably not	234	30.2%
	Don’t know	186	24.0%
How many hours did you expect to wait before seeing a doctor in the ER?	Immediately	163	21.0%
	<1 hour	337	43.4%
	1-4 hours	233	30.0%
	>4 hours	43	5.5%
Have you given any medication/tried any treatment at home for your baby’s illness or injury?	Yes	500	64.4%
	No	276	35.6%
Did you get advice from anyone before coming to the ER?	Yes	426	54.9%
	No	350	45.1%
If yes, who gave the advice?	Relative, friend, or neighbor	204	47.9%
	Baby’s usual doctor	71	16.7%
	Office staff or nurse at doctor’s	51	12.0%
	Pharmacist	50	11.7%
	Telehealth	25	5.9%
	ER staff	16	3.8%
	Doctor	1	0.2%
	Doctor from family	1	0.2%
	Doctors from my family	1	0.2%
	Family doctor	1	0.2%
	Father	1	0.2%
	Grandma gave me advice	1	0.2%
	My brother is a pediatrician	1	0.2%
	937	1	0.2%
	A doctor close to the family	1	0.2%
What was the advice given to you?	Wait and watch baby to see if the condition gets worse	122	28.6%
	I was told to bring the baby to the ER	108	25.4%
	Treat baby at home	74	17.4%
	Call usual doctor	71	16.7%
	I was given an appointment with a doctor for today	36	8.5%
	I was given an appointment with a doctor for one day from now	15	3.5%

**Figure 2:**
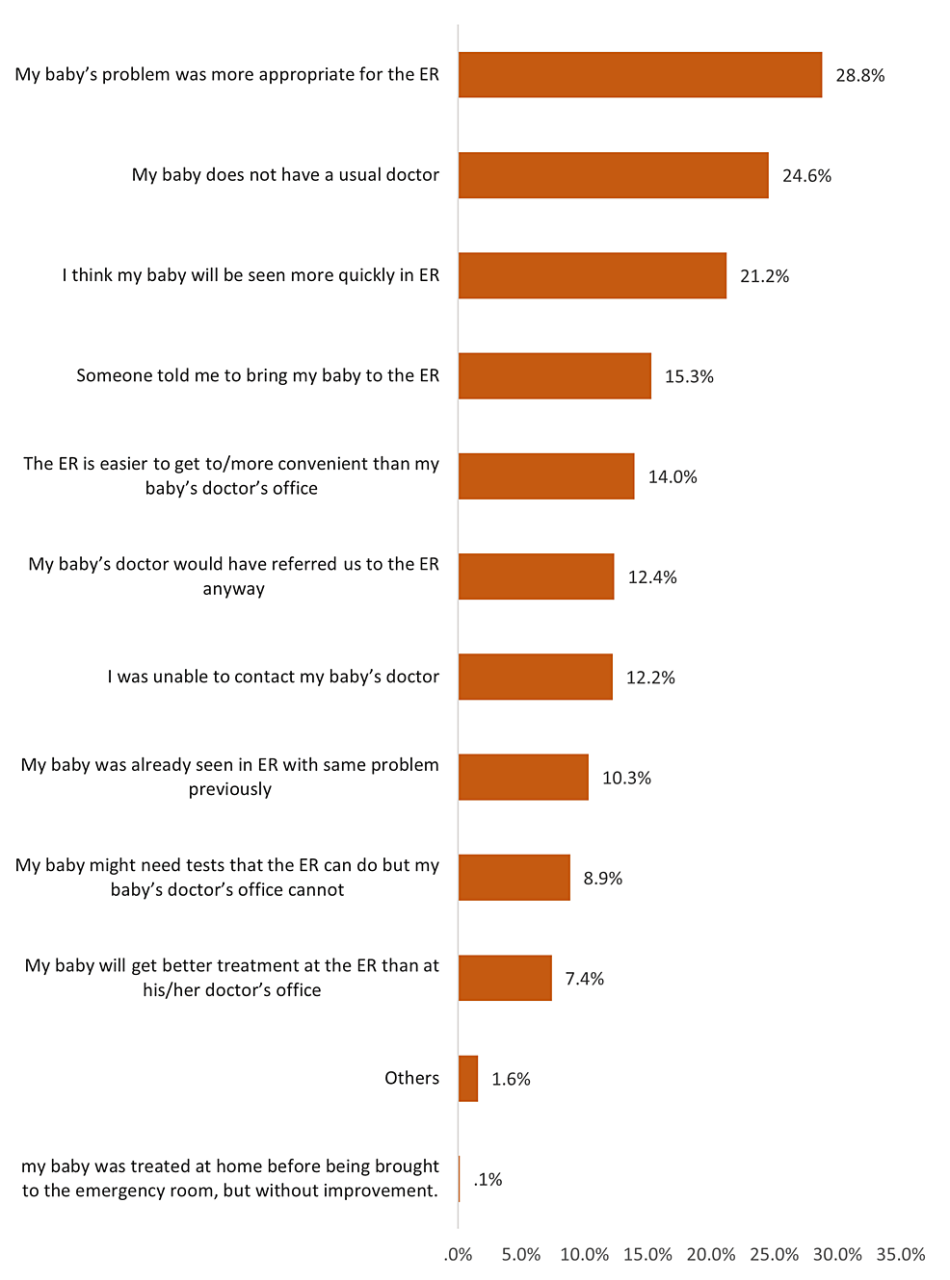
Reasons that made parents decide to go to the ED, Eastern Region, Saudi Arabia

Figure 2 shows the reasons parents decided to go to the ED in the Eastern Region of Saudi Arabia. The most reported reasons were as follows: the baby’s problem could be more appropriately solved in the ED (28.8%), the baby did not have a usual doctor (24.6%), the baby would be seen more quickly in the ED (21.2%), someone told the parents to bring the baby to the ED (15.3%), the ED was easier to get to/more convenient than the baby’s doctor’s office (14%), the baby’s doctor would have referred the parents to the ED anyway (12.4%), and an inability to contact the baby’s doctor (12.2%).

**Figure 3:**
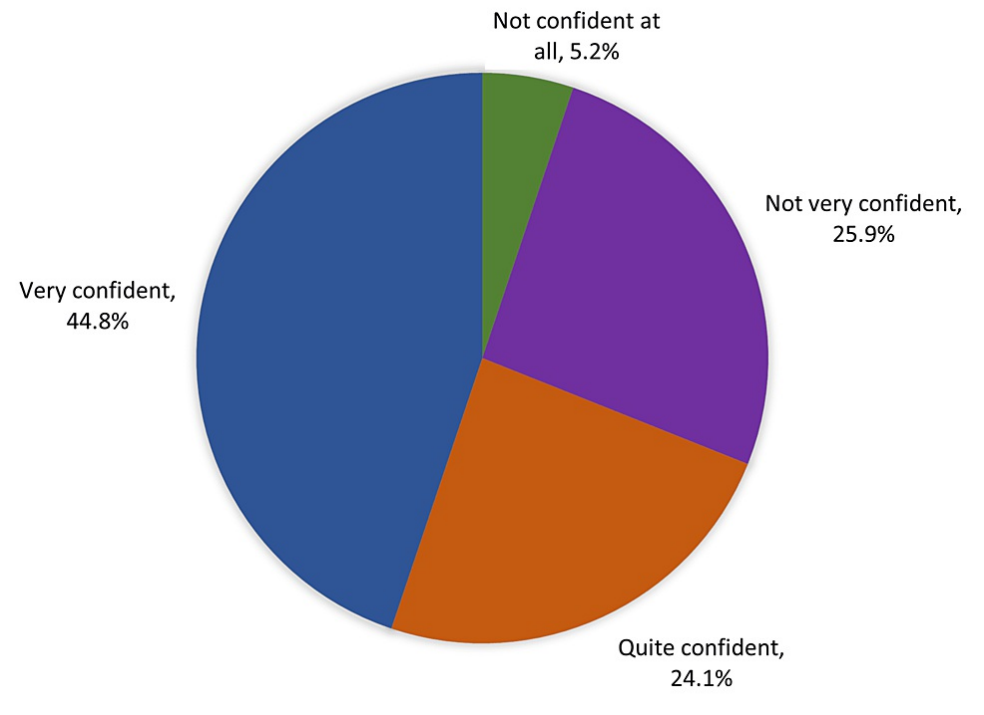
Degree of parents' confidence to look after a child who is sick/injured, Eastern Region, Saudi Arabia

Figure 3 shows the degree of parents’ confidence about looking after a child who was sick/injured in the Eastern Region of Saudi Arabia. A total of 44.8% were very confident, 24.1% were quite confident, and 5.2% were not confident at all.

Table 4 shows the relationship between babies’ conditions and factors influencing ED visits. A total of 28.5% of those who had serious conditions visited the ED from 11 pm to 7 am compared to 18.1% of those who had no serious conditions. The recorded statistical significance was P=0.001. Further, 35.5% of babies with serious conditions were accompanied by both parents versus 42.3% of others (P=0.001). A total of 45.1% of mothers with babies who had serious conditions were not confident about dealing with them in comparison to 24% of parents with babies with nonserious conditions (P=0.001).

**Table 4:** Relationship between babies' condition and factors influencing ED visit P: Pearson X2 test, ^ exact probability test, * P<0.05 (significant)

Factors	To what extent was your baby’s problem serious?						p-value
	Not very serious		Serious		Not sure		
	No	%	No	%	No	%	
Child gender							0.394
Male	162	43.1%	106	43.8%	78	49.4%	
Female	214	56.9%	136	56.2%	80	50.6%	
What time was it when you visited the ED?							0.001*
7 am - 3 pm	130	34.6%	66	27.3%	37	23.4%	
3 pm - 11 pm	178	47.3%	107	44.2%	62	39.2%	
11 pm - 7 am	68	18.1%	69	28.5%	59	37.3%	
Who was with the baby in the ED?							0.001*^
Brother	0	0.0%	1	.4%	3	1.9%	
Father only "	89	23.7%	52	21.5%	41	25.9%	
Grandfather or grandmother "	21	5.6%	39	16.1%	10	6.3%	
Mother and brother	2	0.5%	1	0.4%	0	0.0%	
Mother and uncle	0	0.0%	1	0.4%	0	0.0%	
Mother only "	104	27.7%	61	25.2%	49	31.0%	
Parents together "	159	42.3%	86	35.5%	55	34.8%	
Sister	1	0.3%	1	0.4%	0	0.0%	
How confident are you to look after a child who is sick/injured?							0.001*^
Not confident at all	16	4.3%	14	5.8%	10	6.3%	
Not very confident	74	19.7%	95	39.3%	32	20.3%	
Quite confident	90	23.9%	61	25.2%	36	22.8%	
Very confident	196	52.1%	72	29.8%	80	50.6%	

## Discussion

The ED of a hospital is a crucial part of the healthcare system [17]. The ED is a safety net for the uninsured. With the Emergency Medical Treatment and Labor Act, access to emergency care, irrespective of the ability to pay, has been ensured [18]. The Centers for Disease Control and Prevention collects annual health data, including on the use of the ED. According to the 2014 National Hospital Ambulatory Medical Care Survey summary data, ED visits in the United States totaled more than 141 million, and approximately 30 million of these visits were from pediatric patients younger than 15 years [19,20]. Medicaid, the Children’s Health Insurance Program, or other state-based programs accounted for 35% of ED visits, private insurance or other coverage accounted for approximately 35% of ED visits, and uninsured or self-pay accounted for 12% of ED visits [21].

The current study aimed to determine factors influencing visits to the PED in Saudi Arabia. The results showed that most children visited the ED starting from their first month of life to the age of 10 years. These were also mainly female children. Most of these visits were conducted in the evening, not at night, which is the usual period for seeking emergency childcare. Fever, congestion, and vomiting were the main reasons for visiting the ED. The most reported reason for visiting the ED was parents’ belief that their baby’s problems could be more appropriately solved in the ED. Another reason was that their children had no usual doctor. One-fifth of parents thought that their babies would be seen quickly in the ED, one-fifth expected to be seen immediately in the ED, and less than half expected to wait for less than an hour.

Regarding factors that influenced visits to the PED, about one-half of the parents reported that their child’s problem was not very serious, and less than one-third reported that it was serious. Next, one-third of parents thought that they could wait for 24 hours. This was consistent with Long et al. [1]. In that study, almost half of the parents surveyed stated that their PCP did not offer after-hours or weekend availability, and most did not consider their child’s condition to be serious enough to require immediate care. Additionally, almost 50% of the parents would have waited to see their PCP if they could do so within 24 hours. More than half of the current study’s participants tried medication and treatment at home for their baby’s illness or injury. More than half of them received advice before coming to the ED from a relative, friend, or neighbor; the baby’s usual doctor; office staff or a nurse at the doctor’s office; and the pharmacist (11.7%). The most common advice was to wait and watch the baby to see if the baby’s condition grew worse. Some parents were advised to treat the baby at home. Only one-fourth of parents were advised to bring the baby to the ED, and less than one percent were advised to call their usual doctor. This indicates a lack of awareness about the importance of ED care. In Berry et al.’s [22] study, caregivers cited several reasons for preferring the ED over their child’s PCP. These included long wait times for appointments; dissatisfaction with the PCP; communication issues (e.g., language barriers and unhelpful staff at the PCP’s office); referrals from other healthcare providers; superior efficiency at the ED; and availability of resources, convenience, quality of care, and expertise in treating children. Some parents also expressed a desire for better education on identifying the urgency of pediatric problems. Increased parental ED utilization, single parenthood, Medicaid coverage, and lack of PCP were found to be associated with higher ED utilization in some studies [23-25]. Pehlivanturk-Kizilkan et al. [26] documented that about two-thirds of PED visits were nonurgent. The probability of overestimating an emergency’s severity was higher if the mother was younger, if the mother did not have an income, and if the father had a lower level of education. A systematic review by Conlon et al. [27] highlighted parents’ perception of the severity of their child’s illness, parents’ request for an onward referral, and GPs’ appraisal of parents’ ability to cope. Socioeconomic status, GPs’ aversion to risk, and system-level factors such as access to diagnostics and specialist services also influenced referral decisions. Other studies showed that parents may opt for EDs instead of primary care services because of their perceived urgency and higher quality of care [28,29]. In Saudi Arabia, Almutlaq and Alsuliman [30] documented that the majority of ED cases involved females, making up 56% of the total. The most common reason for visits to the PED was an upper respiratory tract infection, accounting for 55.1% of cases. Only 12.2% of patients had previously visited a primary healthcare center. Interestingly, many of the participants had sources of medical advice other than emergency physicians (80.3%). The results also revealed that 76.2% of parents who brought their children to the ED were not aware that their cases could have been managed in a primary healthcare center.

Limitation

There were several limitations to this descriptive, observational study. First, the parents' judgment of the factors influencing an ED visit was not comparable to the evaluation made by a healthcare provider. Second, our study was unable to address the question of the degree of agreement between the judgments made by parents and medical professionals regarding the severity of their child's condition. Lastly, it's possible that the findings cannot be applied to other states or nations with distinct healthcare systems or demographic profiles. This study, despite its limitations, identifies areas where our present medical system should be improved, as well as community healthcare providers and patient education regarding the proper use of PEDs.

## Conclusions

The current study showed that most PED visits were for nonserious cases, mainly because of parents’ confidence in ED-provided care quality and the expectation of lower waiting times. Additionally, many parents preferred to take their children to the ED whenever they exhibited any symptoms. Physicians should educate families about the advantages of continuity of care for pediatric patients. It can be difficult for primary healthcare staff to provide a medical home for children if parents do not understand that nonurgent ED visits may interrupt the continuity of care. This can have consequences for the child’s overall health. It is also important to discuss the reasons why ED visits may not always be necessary and to clarify when it is appropriate to seek care in the ED.
